# The intermediate filament protein nestin serves as a molecular hub for smooth muscle cytoskeletal signaling

**DOI:** 10.1186/s12931-023-02473-8

**Published:** 2023-06-14

**Authors:** Yinna Wang, Guoning Liao, Yidi Wu, Ruping Wang, Dale D. Tang

**Affiliations:** Department of Molecular and Cellular Physiology, Albany Medical College, 47 New Scotland Avenue, MC-8, Albany, NY 12208 USA

**Keywords:** Smooth muscle contraction, Actin, Intermediate filaments, Protein phosphorylation

## Abstract

**Background:**

The recruitment of the actin-regulatory proteins cortactin and profilin-1 (Pfn-1) to the membrane is important for the regulation of actin cytoskeletal reorganization and smooth muscle contraction. Polo-like kinase 1 (Plk1) and the type III intermediate filament protein vimentin are involved in smooth muscle contraction. Regulation of complex cytoskeletal signaling is not entirely elucidated. The aim of this study was to evaluate the role of nestin (a type VI intermediate filament protein) in cytoskeletal signaling in airway smooth muscle.

**Methods:**

Nestin expression in human airway smooth muscle (HASM) was knocked down by specific shRNA or siRNA. The effects of nestin knockdown (KD) on the recruitment of cortactin and Pfn-1, actin polymerization, myosin light chain (MLC) phosphorylation, and contraction were evaluated by cellular and physiological approaches. Moreover, we assessed the effects of non-phosphorylatable nestin mutant on these biological processes.

**Results:**

Nestin KD reduced the recruitment of cortactin and Pfn-1, actin polymerization, and HASM contraction without affecting MLC phosphorylation. Moreover, contractile stimulation enhanced nestin phosphorylation at Thr-315 and the interaction of nestin with Plk1. Nestin KD also diminished phosphorylation of Plk1 and vimentin. The expression of T315A nestin mutant (alanine substitution at Thr-315) reduced the recruitment of cortactin and Pfn-1, actin polymerization, and HASM contraction without affecting MLC phosphorylation. Furthermore, Plk1 KD diminished nestin phosphorylation at this residue.

**Conclusions:**

Nestin is an essential macromolecule that regulates actin cytoskeletal signaling via Plk1 in smooth muscle. Plk1 and nestin form an activation loop during contractile stimulation.

## Introduction

Airway smooth muscle contraction plays an important role in regulating bronchomotor tone, airway caliber, and ventilation distribution in the lungs. Smooth muscle contraction is regulated by two major pathways: myosin activation and actin polymerization. In response to contractile stimulation, 20-kDa myosin light chain (MLC) undergoes phosphorylation, which promotes crossbridge cycling and myofilament sliding. In addition, a pool of actin monomers incorporate into existing actin filaments, which facilitates smooth muscle contractility by increasing force transmission between the inside and outside of the cells [1–4], and enhancing the quantities of the contractile units [3, 5, 6]. Furthermore, the type III intermediate filament protein vimentin gets phosphorylated, which enhances contractility of smooth muscle and endothelial cells by affecting redistribution of signaling macromolecules [7–10].

Nestin is a type VI intermediate filament (IF) protein that has been implicated in progenitor cell functions, development of nerve systems, cancer pathogenesis, and smooth muscle cell migration [11–13]. Structural analysis reveals that nestin consists of an unusually long C terminal region and short N terminus, which may be associated with the inability to self-form into filaments [14]. Nestin co-assembles with the type III IF protein vimentin in endothelial cells and other cell types [15]. Nestin undergoes phosphorylation at Thr-315 in cancer cells and is associated with cell proliferation [16]. Nevertheless, the role of nestin in smooth muscle contraction has not been previously investigated.

Contractile stimulation induces the recruitment of cortactin and profilin-1 (Pfn-1) to the membrane in smooth muscle cells, which facilitates cortical actin polymerization and force transmission between the contractile units and the extracellular matrix [3, 17]. Cortactin is an adapter protein that regulates actin filament assembly and branching, adhesion, migration, endocytosis, and smooth muscle contraction [17–19]. Cortactin promotes actin polymerization and branching by activating neuronal Wiskott—Aldrich syndrome Protein (N-WASP) and the actin-related protein 2/3 (ARP2/3) complex [18, 20]. Pfn-1 is an actin-binding protein that enhances actin polymerization in vitro [21] and smooth muscle contraction [22]. Pfn-1 facilitates actin filament assembly by catalyzing the exchange of actin-bound ADP for ATP, and by releasing G-actin from thymosin-β4 [23–25].

Polo-like kinase 1 (Plk1) is a serine/threonine protein kinase that participates in the regulation of smooth muscle contraction [8]. Knockdown (KD) of Plk1 reduces the contractile responses of smooth muscle [8, 26]. Plk1 modulates smooth muscle contraction in part by affecting vimentin phosphorylation at Ser-56 [8, 26].

In this study, we evaluated the role and mechanism of nestin in actin cytoskeletal signaling and smooth muscle contractility during agonist stimulation.

## Materials and methods

### Cell culture

Human airway smooth muscle (HASM) cells were prepared from human bronchi and adjacent tracheas obtained from the International Institute for Advanced Medicine [17, 27–32], with studies herein approved by the Albany Medical College Committee on Research Involving Human Subjects. The donor human lungs used to procure tissue and cells were not suitable for transplant, and not identifiable, thus studies were determined to be *Not Human Subjects Research*. Smooth muscle cells within passage 5 were used for the studies [33–35]. The purity of HASM cells was determined by immunostaining for smooth muscle α-actin. Nearly 100% of these cells expressed α-actin [35]. Primary cells from three non-asthmatic donors were used for the experiments. In some cases, duplicate or triplicate experiments from cells of one donor were used for analysis. HASM cells were serum starved for 24 h before experiments.

### Immunoblot analysis

Western blotting of cell lysis was performed using the experimental procedures as previously described [8, 28, 35–37]. Antibodies used were anti-nestin (1:1000, Fisher Invitrogen #PIPA511887/L/N SH2420723H and Cell Signaling #10959s/L/N 1), anti-GAPDH (1: 1000, Santa Cruz Biotechnology #sc-32233/L/N K0315), anti phospho-myosin light chain (Ser-19, Santa Cruz Biotechnology, 1:500), anti-myosin light chain (1:1000, custom made) [8, 38, 39], anti-vimentin (BD Biosciences, #550515, L/N 3214517, 1:10,000), anti-phospho-vimentin (Ser-56) (custom made) [8, 40, 41], anti-Plk1 (1:1000, EMD Millipore, #05-844, L/N 2477015), anti-phospho-Plk1 (T210) (Cell Signaling 9062 S1), and anti-phospho-nestin (Thr-315) (Santa Cruz Biotechnology, 1:500, sc-377538, J2820).

The antibodies were validated by examining the molecular weight of target proteins. In addition, anti-nestin and anti-Plk1 were validated by using KD cells. Finally, vendors have provided datasheet to show that antibodies were validated by positive controls. The levels of proteins were quantified by scanning densitometry of immunoblots (Fuji Multi Gauge Software or GE IQTL software). The luminescent signals from all immunoblots were within the linear range.

### Co-immunoprecipitation analysis

Protein–protein interactions were evaluated by co-immunoprecipitation analysis as previously described [8, 28, 35, 36, 42] with minor modification. Briefly, cell extracts were incubated overnight with corresponding antibodies and then incubated for 3 h with 20 µl of the Protein A/G Plus-Agarose reagent (Santa Cruz Biotechnology). Immunocomplexes were washed four times in buffer containing 50 mM Tris–HCl (pH 7.6), 150 mM NaCl and 0.1% Triton X-100. The immunoprecipitates were separated by SDS-PAGE followed by transfer to nitrocellulose membranes. The membranes of immunoprecipitates were probed with use of corresponding antibodies.

### Lentiviral transduction

Stable KD cells were generated using lentiviruses encoding target shRNA as previously described. [27, 43]. Briefly, lentiviruses encoding nestin shRNA (sc-36032-V) and control shRNA (sc-108080) were purchased from Santa Cruz Biotechnology. Human ASM cells were infected with control shRNA lentiviruses or target shRNA lentiviruses for 12 h followed by 3–4 day culture, and selected with puromycin to generate positive clones expressing shRNAs. The expression levels of nestin were assessed by immunoblot analysis.

Stable Plk1 KD HASM cells were generated as previously described [8]. These KD cells and cells expressing control shRNA were stable for at least five passages after initial infection.

### Plasmids and cell transfection

Human nestin cDNA was purchased from Genomics Online Inc (pENTR223-1-human nestin cDNA, catalog number ABIN3427596, nestin cDNA NCBI Accession number GU014842). pLenti-puro mammalian expression vector containing a pCMV promoter was purchased from Addgene (Plasmid # 39481). We subcloned nestin cDNA into the pLenti-puro vector through Nhe I/Spe I and EcoR V sites. Cells were transfected with the constructs encoding WT or mutant nestin using the FuGene HD transfection reagent according to the instruction of the manufacture (Promega).

### Immunofluorescence microscopy

Immunostaining of cells were performed using similar methods as previously described [27, 28, 41, 43–46]. Briefly, cells were fixed for 15 min in 4% paraformaldehyde, and were then washed three times in phosphate-buffered saline (PBS) followed by permeabilization with 0.2% Triton X-100 dissolved in PBS for 5 min. These cells were immunofluorescently stained using specific antibodies followed by appropriate secondary antibodies conjugated with fluorophores (Invitrogen). The cellular localization of fluorescently labeled proteins was viewed under a high resolution digital fluorescent microscopy (Leica, 63 × oil objective). The localization of labeled proteins was also line scanned using Leica Image software. Image analysis for protein localization was performed using the previously-described method with minor modification [38, 47]. By using Leica DMI 6000 software, the pixel intensity was quantified for minimal five line scans across the periphery of cells (nuclei excluded). Ratios of pixel intensity at the cell edge to pixel intensity at the cell interior were determined for each line scan as follows: ratios of the average maximal pixel intensity at the cell periphery to minimal pixel intensity in the cell interior. The ratios of pixel intensity at the cell border to that in the cell interior for all the line scans performed on a given cell were averaged to obtain a single value for the ratio of each cell.

### Analysis of F-actin/G-actin ratios

The content of F-actin and G-actin in smooth muscle cells was evaluated using the actin fractionation assay as previously [36, 38, 39, 48].

### Site-directed mutagenesis

Wild type nestin was used as template to generate T315A nestin mutant using the Quick change II XL site-directed mutagenesis kit (Agilent Technologies) as previously described [31, 49]. The sequence of forward primer was 5’-GAA CTC CCG GCT GCA AGC ACC TGG CGG TGG CTC C-3’. The sequence of reverse primer was 5’-GGA GCC ACC GCC AGG TGC TTG CAG CCG GGA GTT C-3’. Plasmids were purified by using the QIAPrep Spin Miniprep kit (Qiagen, Germany). DNA sequencing was performed by Azenta.

### Precision cut lung slices (PCLS)

Human lungs were obtained from the International Institute for Advanced Medicine [17, 27–30, 38]. Again, human tissues were non-transplantable, and informed consents were obtained from all subjects for research. This study was approved by the Albany Medical College Committee on Research Involving Human Subjects. Bronchi (Generation 2–3) were slowly filled with warm 2% low-melting-point agarose (type VII) prepared in Hank’s Balanced Salt Solution (HBSS). Once the lungs were filled with agarose, bronchi were clamped to prevent leakage. Lungs were rinsed with ice-cold HBSS supplemented with HEPES and placed at 4 °C overnight to allow the agarose to cool completely. Cubes (1 to 1.5-cm) of lung tissue were prepared and cut transversely at 300 µm in HBSS supplemented with HEPES using a Vibratome (LeicaVT1200S). The PCLS were placed in warm Dulbecco’s Modified Eagle Medium (DMEM) supplemented with antibiotics and incubated at 37 °C with 5% CO_2_. Prior to experiments, PCLS were placed in the modified physiological saline solution (PSS; 110 mM NaCl, 3.4 mM KCl, 0.8 mM MgSO4, 4.8 mM CaCl_2_, 25 mM HEPES, 1 g/L dextrose, pH 7.4) for 30 min in an incubator and subsequently placed in a 35-mm glass bottom culture dish with the modified physiological saline solution. PCLS were treated with 100 µM acetylcholine (ACh), and images of airway lumen were captured using a time-lapse microscopy (Leica DMI 6000, 10 × object, phase-contrast). Change in lumen area was examined relative to that induced by stimulation of PCLS with ACh. For tissue transfection, PCLS were transfected with control siRNA (Santa Cruz Biotechnology, SC-37007/C1522) nestin siRNA (Santa Cruz Biotechnology, SC-36032/F1213), the constructs encoding WT or mutant nestin premixed with the FuGene HD transfection reagent according to the instruction of the manufacture (Promega) [38].

### Statistical analysis

All statistical analysis was performed using Prism software (GraphPad Software, San Diego, CA). Differences between pairs of groups were analyzed by Student’s *t*-test. Comparison among multiple groups was performed by one-way or two-way ANOVA followed by a post hoc test (Tukey’s multiple comparisons). Values of n refer to the number of experiments used to obtain each value. P < 0.05 was considered to be significant.

## Results

### Role of nestin in actin polymerization and MLC phosphorylation in smooth muscle

**Fig. 1:**
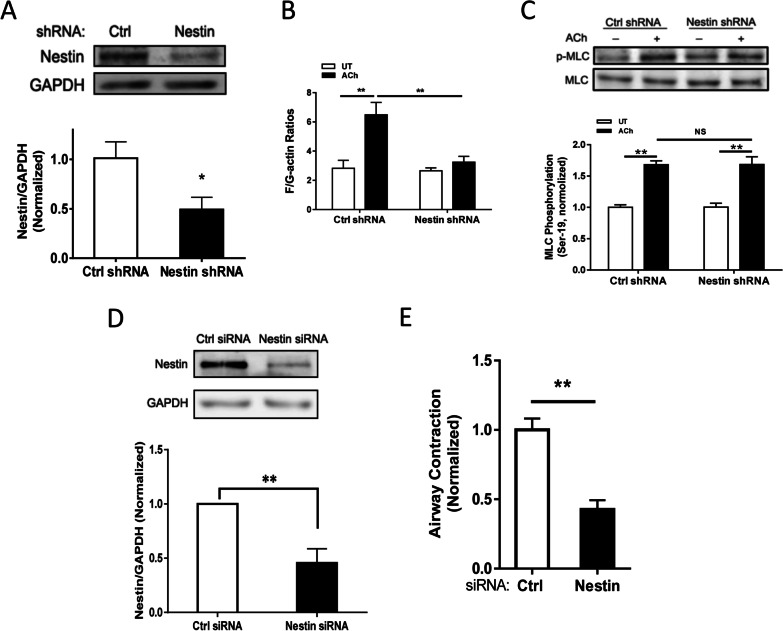
Nestin regulates actin polymerization and smooth muscle contraction without affecting myosin light chain phosphorylation at Ser-19. **A** Protein expression of human airway smooth muscle (HASM) cells expressing control (Ctrl) or nestin shRNA was evaluated by immunoblot analysis. Nestin shRNA reduces nestin expression in HASM cells. Data are means ± SE (n = 4 batches of culture from 3 donors). **B** Ctrl and nestin knockdown (KD) HASM cells were treated with 10^–4^ M acetylcholine (ACh) for 5 min, or left untreated (UT). F-actin/G-actin ratios in cells were evaluated using the fractionation assay. Data are means ± SE (n = 5 batches of culture from 3 donors). **C** Myosin light chain (MLC) phosphorylation at Ser-19 in Ctrl cells and nestin KD cells was assessed by immunoblot analysis. Basal and ACh-induced MLC phosphorylation was similar in Ctrl cells and nestin KD cells (NS, not significant). Data are means ± SE (n = 4–5 batches of culture from 3 donors). **D** Immunoblots showing the effects of nestin siRNA on protein expression in human PLCS. Data are means ± SE (n = 3 slices from 3 donors). **E** Nestin KD reduces airway constriction of human PLCS. Data are means ± SE (n = 4–6 slices from 3 donors). *p < 0.05; **p < 0.01. Student’s *t*-test was used for statistical analysis of **A**, **D** and **E**. Two-way ANOVA was used for statistical analysis of **B** and **C**

To assess the functional role of nestin, we generated stable nestin KD cells by using lentivirus encoding nestin shRNA. Immunoblot analysis showed that nestin expression was reduced by approximately 50% in cells transduced with nestin shRNA (Fig. 1A). Because nestin is involved in cell growth [12], complete KD of nestin impairs cell viability. Thus, we used the experimental condition, in which nestin was partially downregulated, for the following experiments.

Because both actin polymerization and MLC phosphorylation are important for smooth muscle contraction [2–4, 8, 50, 51], we assessed the effects of nestin KD on the two cellular processes. HASM cells were stimulated with ACh or left unstimulated. F/G-actin ratios and MLC phosphorylation were determined by the actin fractionation assay and immunoblot analysis, respectively. Nestin KD attenuated F/G-actin ratios (an indication of actin polymerization) upon ACh stimulation without affecting MLC phosphorylation (Fig. 1B, C). These studies unveil a new role for nestin in regulating the actin cytoskeleton in smooth muscle.

### Nestin regulates airway smooth muscle contraction

Nestin is involved in the regulation of self-renewal and differentiation of stem cells [11] and migration of smooth muscle cells [13]. The role of nestin in smooth muscle contraction has not been previously investigated. We have established the technique to determine airway narrowing of PCLS [38, 52]. We treated human PCLS with control or nestin siRNA. Immunoblot analysis was used to assess protein expression. The expression of nestin protein was reduced in lung slices treated with nestin siRNA (Fig. 1D, top panel). We then assessed intrapulmonary airway constriction. Airway constriction in PCLS was reduced by nestin KD (Fig. 1D, bottom panel).

### Role of nestin in recruitment of cortactin and Pfn-1 to cell periphery upon contractile stimulation

**Fig. 2:**
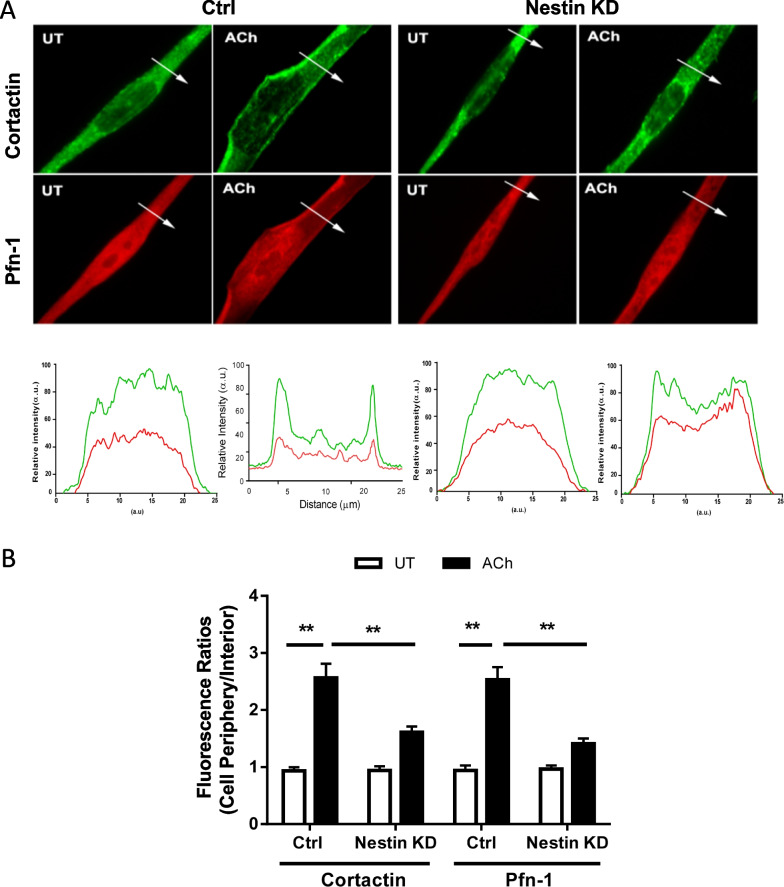
Nestin modulates the recruitment of cortactin and Pfn-1 to the membrane. Ctrl and nestin knockdown (KD) HASM cells were treated with 10^–4^ M ACh for 5 min, or left untreated (UT). Spatial distribution of cortactin and Pfn-1 was evaluated by immunfluorescence microscopy. **A** Representative micrographs illustrating the effects of ACh on the spatial localization of cortactin and Pfn-1 in HASM cells. The arrows indicate a single line scan to quantify the fluorescent signals in cells. The line scan graphs show relative fluorescent intensity. **B** Image analysis for protein localization was described in the section of Materials and Methods. Protein distribution in cells is expressed as ratio of the intensity at the cell periphery to cell interior. Data are means ± SE (n = 6 batches of culture from 3 donors). *p < 0.05; ** *p* < 0.01

Actin-regulatory proteins including cortactin and Pfn-1 are recruited to the membrane upon contractile activation, which regulates cortical actin polymerization [17, 36]. Thus, we tested whether nestin regulates the recruitment of these proteins to the cell edge. We used immunofluorence microscopy to investigate the spatial distribution of cortactin and Pfn-1 after contractile activation. In cells not treated with ACh, cortactin and Pfn-1 largely localized in the myoplasm. In contrast, cortactin and Pfn-1 mainly localized on the cell periphery of cells treated with ACh (Fig. 2).

### Contractile stimulation induces nestin phosphorylation at Thr-315 in smooth muscle cells

**Fig. 3:**
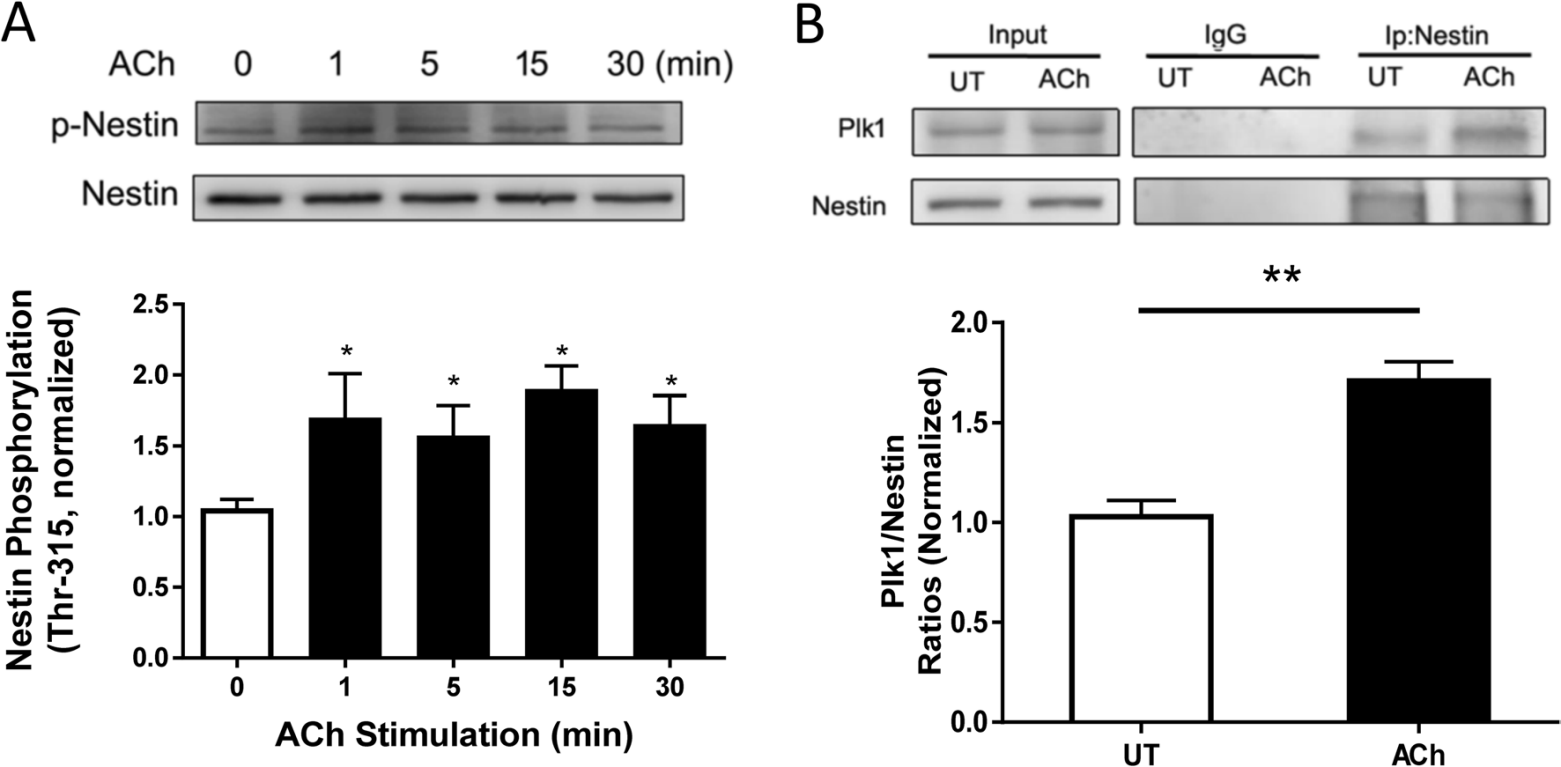
Contractile stimulation enhances nestin phosphorylation at Thr-315 and nestin/Plk1 coupling. HASM cells were treated with 10^–4^ M ACh for different time points, or left untreated (UT). **A** Nestin phosphorylation at Thr-315 was determined by immunoblot analysis. Data are means ± SE (n = 5 batches of culture from 3 donors). **B** ACh stimulation enhances the ratios of Plk1/immunoprecipitated nestin without affecting protein expression in input fraction. IgG serves as a control. Data are means ± SE (n = 4 batches of culture from 3 donors). *p < 0.05; ** p < 0.01

Because nestin gets phosphorylated at Thr-315 in cancer cells during proliferative response [16], we questioned whether contractile activation induces nestin phosphorylation at this residue. HASM cells were stimulated with ACh for different time points, or left unstimulated. Nestin phosphorylation in these cells was evaluated by immunoblot analysis using nestin phosphorylation site-specific antibody. Nestin phosphorylation at Thr-315 was increased as early as 1 min after ACh stimulation, and sustained at elevated level 30 min after ACh stimulation (Fig. 3A).

### The interaction of nestin with Plk1 is regulated by contractile activation

Because both nestin and Plk1 play important roles in regulating smooth muscle contraction, we questioned whether contractile activation affects the association of nestin with Plk1. HASM cells were treated with ACh or left untreated. Extracts of these cells were immunoprecipitated with nestin antibody and immunoblotted with antibodies against nestin and Plk1. Coprecipitated Plk1 in stimulated cells was higher than control cells. Ratios of Plk1 over precipitated nestin were greater in ACh-treated cells than in untreated cells (Fig. 3B).

### Nestin orchestrates phosphorylation of Plk1 and vimentin

**Fig. 4:**
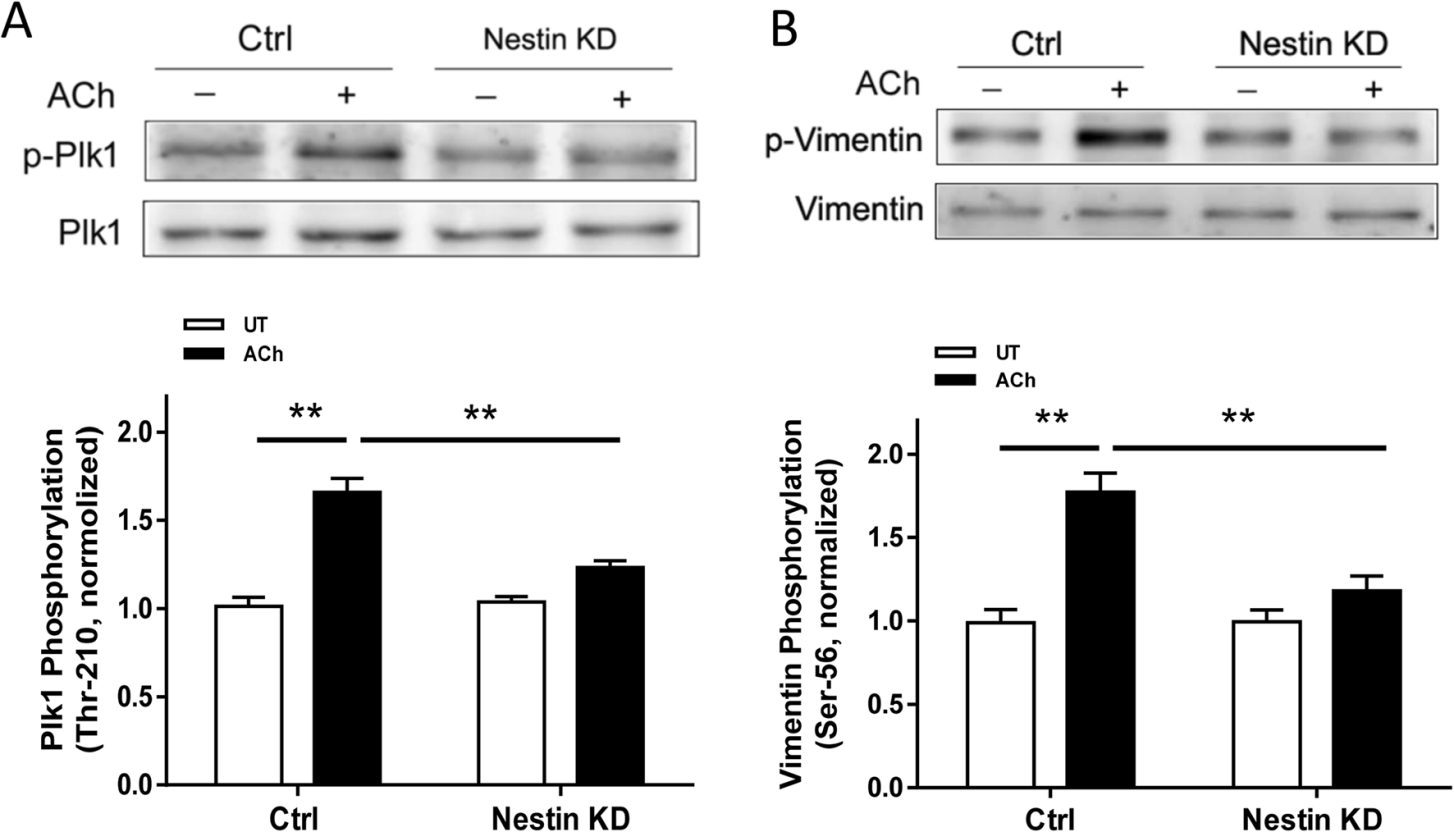
Nestin regulates Plk1 phosphorylation at Thr-210 and vimentin phosphorylation at Ser-56. Ctrl and nestin KD HASM cells were treated with 10^–4^ M ACh for 5 min, or left untreated (UT). Protein phosphorylation in HASM cells was determined by immunoblot analysis. Nestin KD diminishes ACh-induced Plk1 phosphorylation at Thr-210 (**A**) and vimentin phosphorylation at Ser-56 (**B**). Data are means ± SE (n = 4 batches of culture from 3 donors). ** *p* < 0.01

Because nestin interacts with Plk1, we evaluated whether nestin is involved in phosphorylation of Plk1 at Thr-210 (an indication of Plk1 activation) [8] by assessing the effects of nestin KD on Plk1 phosphorylation. The ACh-induced Plk1 phosphorylation at Thr-210 was reduced by nestin KD (Fig. 4A). As described earlier, Plk1 regulates smooth muscle contraction in part by modulating vimentin phosphorylation. We determined the influence of nestin KD on vimentin phosphorylation at Ser-56, an index of vimentin activation [8, 35]. Vimentin phosphorylation at Ser-56 upon ACh stimulation was diminished in nestin KD cells (Fig. 4B).

### Role of nestin phosphorylation at Thr-315 in actin polymerization and MLC phosphorylation and airway contraction

**Fig. 5:**
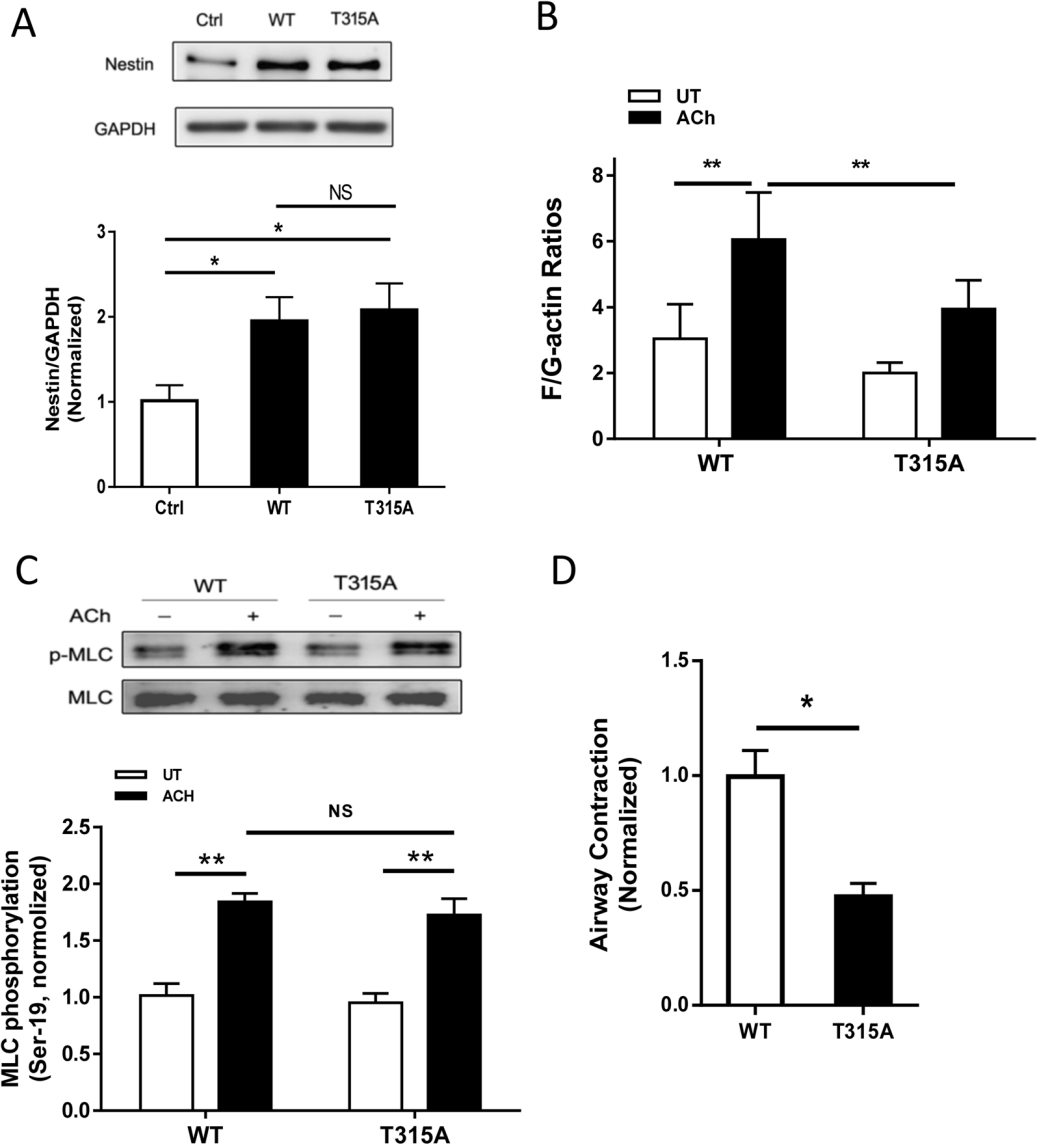
Role of nestin phosphorylation at Thr-315 in actin polymerization, MLC phosphorylation and smooth muscle contraction. **A** Extracts of HASM cells treated with wild type (WT) or T315A nestin were separated by SDS PAGE and immunobotted with antibodies against nestin and GAPDH. Data are means ± SE (n = 4–5 batches of culture from 3 donors). **B** HASM cells expressing WT or T315A nestin were treated with 10^–4^ M ACh for 5 min, or left untreated (UT). F-actin/G-actin ratios in cells were evaluated using the fractionation assay. Data are means ± SE (n = 4–5 batches of culture from 3 donors). **C** MLC phosphorylation at Ser-19 in cells treated with WT or T315A nestin was assessed by immunoblot analysis. Basal and ACh-induced MLC phosphorylation was similar in cells expressing WT and T315A nestin (NS, not significant). Data are means ± SE (n = 4–5 batches of culture from 3 donors). **D** Expression of T315A nestin reduces airway constriction of human PLCS. Data are means ± SE (n = 4–6 slices from 3 donors). *p < 0.05; ** *p* < 0.01. One-way ANOVA was used for statistical analysis of **A** Two-way ANOVA was used for statistical analysis of **B** and **C**. Student’s *t*-test was used for statistical analysis of **D**

Next, we evaluated whether phosphorylation of nestin at Thr-315 is important for the regulation of smooth muscle cytoskeletal reorganization and myosin activation. HASM cells were transfected with wild type (WT) or T315A nestin followed by immunoblot analysis. Expression of WT and T315 nestin was verified in HASM cells (Fig. 5A). The expression of T315A nestin diminished the ACh-enhanced F-actin/G-actin ratios as compared to cells expressing WT nestin (Fig. 5B). However, MLC phosphorylation was not significantly different between cells treated with WT and T315A nestin (Fig. 5C). Furthermore, the treatment with T315A nestin reduced airway constriction in PCLS (Fig. 5D).

### Expression of T315A nestin diminishes spatial redistribution of cortactin and Pfn-1 to cell periphery

**Fig. 6:**
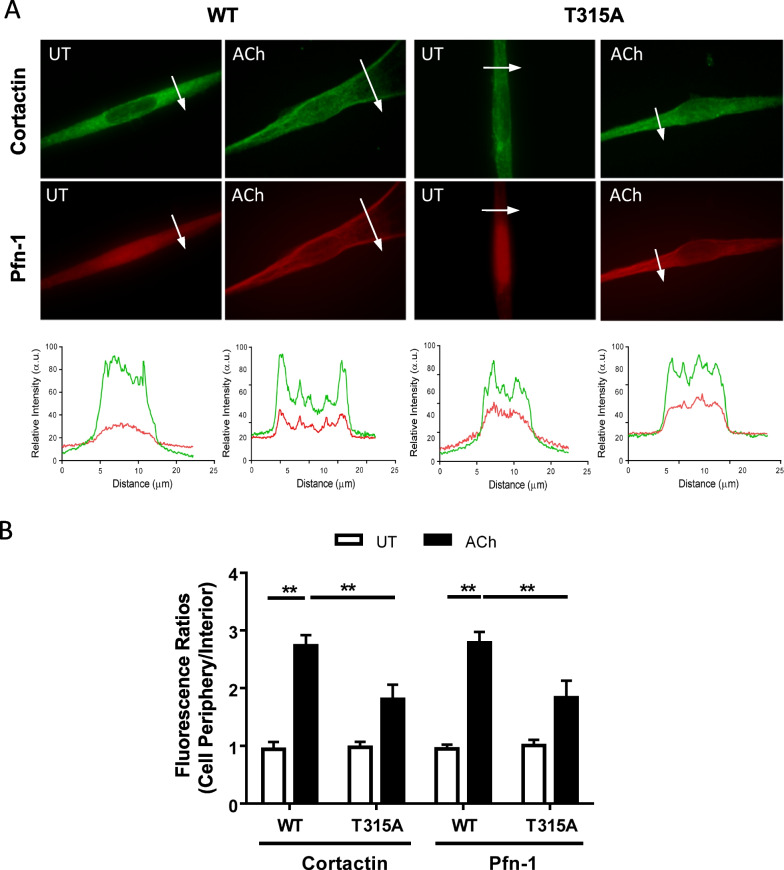
Nestin phosphorylation at Thr-315 orchestrates redistribution of cortactin and Pfn-1 to the membrane. Cells expressing WT or T315A nestin were treated with 10^–4^ M ACh for 5 min, or left untreated (UT). Cellular localization of cortactin and Pfn-1 was evaluated by immunfluorescence microscopy. **A** Representative micrographs illustrating the effects of T315A nestin on the spatial localization of cortactin and Pfn-1 in HASM cells. The arrows indicate a single line scan to quantify the fluorescent signals in cells. The line scan graphs show relative fluorescent intensity. **B** Image analysis for protein localization was described in the section of Materials and Methods. Protein distribution in cells is expressed as ratio of the intensity at the cell periphery to cell interior. Data are means ± SE (n = 6 batches of culture from 3 donors). *p < 0.05; ** *p* < 0.01

We also assessed the role of nestin phosphorylation at this position in protein translocation by using immnofluorescence microscopy. The expression of WT nestin did not affect the redistribution of cortactin and Pfn-1 to the membrane in response to ACh activation. However, the recruitment of cortactin and Pfn-1 to cell periphery was reduced in cells expressing T315A nestin after ACh stimulation (Fig. 6).

### Plk1 regulates nestin phosphorylation at Thr-315

**Fig. 7:**
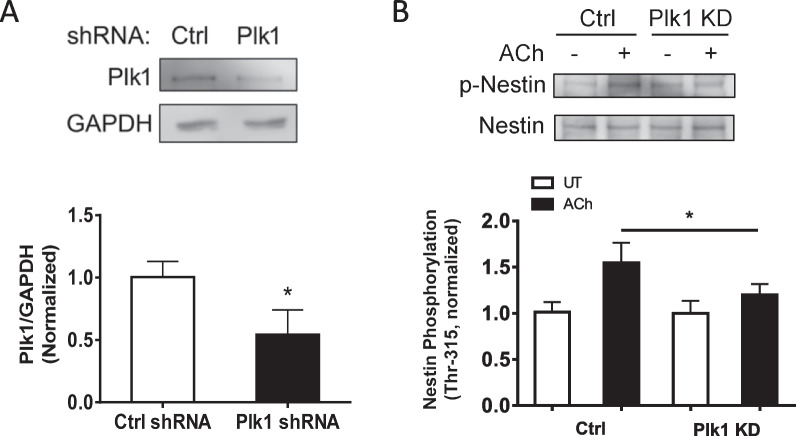
Plk1 regulates nestin phosphorylation at Thr-315 in smooth muscle. **A** Protein expression of Ctrl and Plk1 KD HASM cells were evaluated by immunoblot analysis. **B** Ctrl and Plk1 KD HASM cells were treated with 10^–4^ M ACh for 5 min, or left untreated. Nestin phosphorylation was determined by immunoblot analysis. Plk1 KD inhibits ACh-induced nestin phosphorylation at Thr-315. Data are means ± SE (n = 4–5 batches of culture from 3 donors). * *p* < 0.05

Because contractile stimulation induces nestin phosphorylation and nestin/Plk1 coupling, we questioned whether Plk1 affects nestin phosphorylation. We have generated stable Plk1 KD cells [8] and assessed the effects of Plk1 KD on nestin phosphorylation at this residue. Interestingly, Plk1 KD attenuated the ACh-induced phosphorylation of nestin at Thr-315 (Fig. 7). Because nestin KD also affects Plk1 phosphorylation (Fig. 4A), these results suggest that nestin phosphorylation and Plk1 phosphorylation are interdependent.

## Discussion

Nestin is a type VI IF protein that has been implicated in progenitor cell functions, neuronal development, cancer pathobiology, and smooth muscle cell migration [11–13]. The functional role of nestin in smooth muscle contraction has not been previously investigated. Here, we show that nestin positively regulates airway smooth muscle contraction by modulating the recruitment of cytoskeletal signaling macromolecules and actin cytoskeletal remodeling.

Both myosin activation and actin polymerization are required for smooth muscle contraction. Disruption of either of the two pathways impairs smooth muscle contractile property [1–4]. In this study, nestin KD reduced the agonist-induced actin polymerization and intrapulmonary bronchial constriction without affecting MLC activation. Moreover, c-Abl tyrosine kinase orchestrates the redistribution of cortactin and Pfn-1 to the cell edge, which facilitates cortical actin polymerization in smooth muscle [17]. Here, the recruitment of cortactin and Pfn-1 to the membrane upon contractile activation was also diminished in nestin-deficient cells. The results are intriguing because we identify a new mechanism that modulates the redistribution of these macromolecules, which may promote actin cytoskeletal reorganization and smooth muscle contraction.

Upon external stimulation, cytoskeletal proteins are able to undergo post-translational modifications and alter their structural and biochemical properties. Because nestin gets phosphorylated at Thr-315 in cancer cells and is associated with cell proliferation [16], we questioned whether contractile stimulation affects nestin phosphorylation at this position. Our biochemical analysis demonstrated that ACh stimulation elicited nestin phosphorylation at Thr-315, which was sustained at elevated levels after initial surge. Plk1 is a protein kinase that regulates smooth muscle contraction by affecting the intermediate filament protein vimentin [8]. In this report, contractile stimulation facilitated the coupling of nestin with Plk1 in smooth muscle. Because protein–protein interactions have been implicated in regulating functions of target proteins, we evaluated the role of nestin in Plk1 activation. Nestin KD reduced Plk1 phosphorylation at Thr-210 during contractile activation. Moreover, nestin KD inhibited the agonist-induced vimentin phosphorylation at Ser-56. Vimentin phosphorylation has been shown to regulate redistribution of p130 Crk-associated substrate [8, 9, 47]. Therefore, it is likely that nestin regulates the Plk1-vimentin pathway, which regulates the redistribution of cortactin and Pfn-1, actin dynamics, and smooth muscle contraction. The mechanisms by which vimentin phosphorylation affects translocation of macromolecules are not well understood. It is possible that vimentin phosphorylation increases negative charges on the molecule and induces conformational changes, which facilitates protein redistribution [3, 9, 41]. Although nestin interacts with Plk1 in this study, we do not rule out the possibility that nestin may interact with other macromolecules or Plk1 may associate with other molecules in smooth muscle during contractile stimulation. For instance, protein phosphatase 1 (PP1) and protein phosphatase 2A (PP2A) have been implicated in smooth muscle contraction [7, 53]. PP1 and PP2A serve as catalytic subunits and interact with other regulatory subunits (cofactors) to form holoenzymes for specific substrate dephosphorylation. It is possible that nestin associates with PP1 and/or PP2A, and regulates cytoskeletal signaling in smooth muscle. Future studies are required to test the possibility.

Here, the expression of T315A nestin inhibited the agonist-elicited recruitment of cortactin and Pfn-1, actin polymerization, and contractile response without influencing MLC phosphorylation. The results suggest that nestin phosphorylation at this residue is important to regulate cytoskeletal signaling and smooth muscle contraction.

Contractile stimulation elicits Plk1 phosphorylation at Thr-210 in smooth muscle, which induces conformational changes of the kinase and increases its activity [8]. In this study, we found that Plk1 also orchestrated the phosphorylation of nestin at Thr-315. This finding is very interesting because nestin phosphorylation and Plk1 phosphorylation are interdependent. Because Plk1 undergoes autophosphorylation in smooth muscle [3, 8], it is possible that contractile stimulation initiates Plk1 autophosphorylation, which mediates nestin phosphorylation at Thr-315. Phosphorylated nestin promotes formation of the nestin and Plk1 complex, and renders Plk1 in active state. This activation loop may help cells to sustain its functional state at lower energy cost. Specifically, this activation loop facilitates more efficient actin polymerization and force transmission between the contractile unit and the extracellular matrix.

**Fig. 8:**
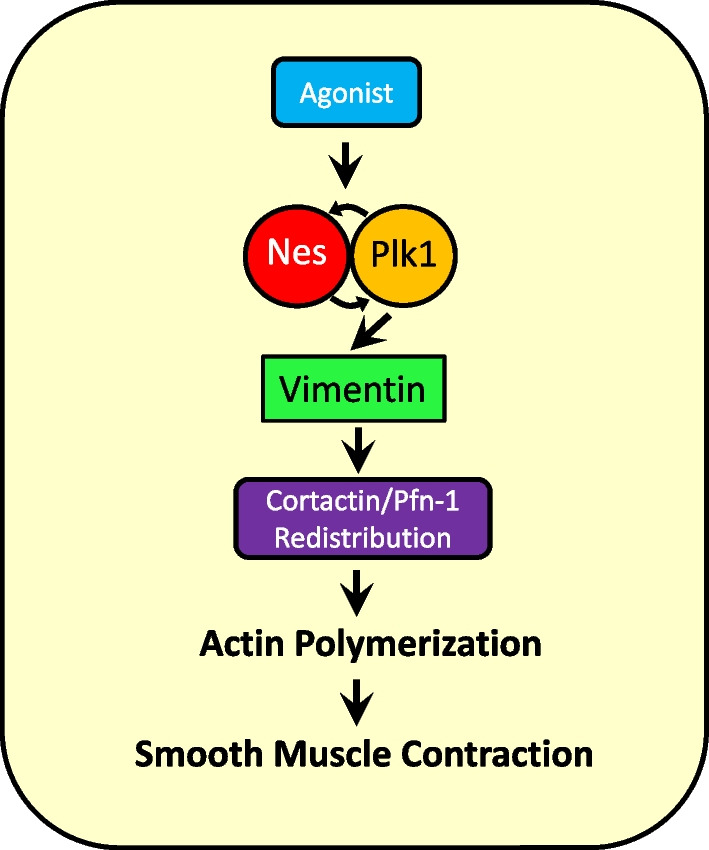
Role of nestin in cytoskeletal signaling. Contractile stimulation promotes the association of nestin with Plk1, which facilitates nestin phosphorylation at Thr-315, and renders Plk1 in active state. This nestin/Plk1 interaction forms a positive activation loop. Activated Plk1 catalyzes vimentin phosphorylation at Ser-56, which subsequently promotes the recruitment of cortactin and Pfn-1 to the membrane, actin polymerization, and smooth muscle contraction

In summary, we unveil a novel mechanism by which nestin regulates cytoskeletal signaling in smooth muscle. Contractile stimulation initiates Plk1 autophosphrylation, which mediates nestin phosphorylation at Thr-315. Phosphorylated nestin facilitates formation of the nestin and Plk1 complex and renders Plk1 in active state. Activated Plk1 catalyzes vimentin phosphorylation at Ser-56, which promotes the recruitment of cortactin and Pfn-1 to the membrane, actin polymerization and smooth muscle contraction (Fig. 8).

## Data Availability

Essential datasets supporting the conclusions are included in this published article.

## Abbreviations

ACh: Acetylcholine
Arp 2/3: Actin-related protein 2/3
GAPDH: Glyceraldehyde-3-phosphate dehydrogenase
HASM: Human airway smooth muscle
IF: Intermediate filament
KD: Knockdown
MLC: Myosin light chain
N-WASP: Neuronal Wiskott—Aldrich syndrome Protein
PCLS: Precision cut lung slices
Pfn-1: Profilin-1
Plk1: Polo-like kinase
shRNA: Short hairpin RNA
siRNA: Small interfering RNA
WT: Wild type
